# Effectiveness of Nursing Documentation Frameworks (SBAR, SOAP, and PIE) in Enhancing Clinical Handoffs and Patient Safety

**DOI:** 10.7759/cureus.89957

**Published:** 2025-08-13

**Authors:** Esthela Carolina Hidalgo Tapia, Joanna León Yosa, María Humbelina Olalla García, Nube Janeth Clavijo Morocho, Yesenia Alexandra Sanmartín Calle

**Affiliations:** 1 Nursing Education, Universidad de Cuenca, Cuenca, ECU; 2 Nursing Education, Universidad Estatal de Bolivar, Guaranda, ECU

**Keywords:** clinical handoffs, nursing documentation, patient safety, pie, sbar, soap, structured communication

## Abstract

Constructed communication models, including SBAR (Situation, Background, Assessment, Recommendation), SOAP (Subjective, Objective, Assessment, Plan), and PIE (Problem, Intervention, Evaluation), have become more and more significant in the field of nursing because of improving handoff communication and ensuring patient safety. The purposes of these documentation formats are to achieve consistency in sharing information, keep to a minimum the occurrence of errors, and enhance the concreteness and effectiveness of communication between healthcare professionals.

The systematic review was conducted to determine how the SBAR, SOAP, and PIE models can help to enhance the quality of the handoff process, alleviating the number of clinical errors and ensuring the safety of patients in different clinical contexts. The review was conducted in accordance with the Preferred Reporting Items for Systematic Reviews and Meta-Analyses (PRISMA) 2020 guidelines. A comprehensive literature search was performed across PubMed, Web of Science, and the Cochrane Library using a combination of text words and controlled vocabulary. Studies published in English between January 2005 and May 2025, involving registered nurses and nursing staff in hospital or inpatient settings, were included. Only quasi-experimental studies, randomized controlled trials, and mixed-method studies were considered. Risk of bias was assessed using the Cochrane Risk of Bias (RoB) 2.0 for randomized controlled trials and the Risk of Bias In Non-randomized Studies of Interventions (ROBINS-I) for non-randomized studies, and the methodological quality of mixed-method studies was appraised with the Mixed Methods Appraisal Tool (MMAT). Risk of bias figures were visualized using the robvis tool.

A total of 15 studies met the inclusion criteria. The quality of the 15 included studies ranged from moderate to high. Findings demonstrated that structured documentation frameworks significantly improve communication clarity, reduce information omissions, enhance documentation accuracy, and lower the incidence of handoff-related errors.

SBAR was particularly effective in high-acuity settings, while SOAP and PIE were beneficial in routine care and ongoing patient assessment. Overall, the implementation of structured nursing documentation enhances interdisciplinary collaboration, supports safer transitions of care, and contributes to improved patient outcomes.

## Introduction and background

Communication in healthcare is critical for ensuring the safety and continuity of care of a patient, particularly during clinical handoffs, which present one of the highest times of potential risk for significant error or adverse patient outcomes [1]. To make the communication standard and enhance the accuracy of imparting the patient information, nursing documentation frameworks like SBAR (Situation, Background, Assessment, Recommendation), SOAP (Subjective, Objective, Assessment, Plan), and PIE (Problem, Intervention, Evaluation) are used widely, especially SBAR, which was introduced by the navy in the early 2000s and implemented in healthcare in the mid-2000s. Such frameworks offer systematic methods of recording and communicating patient information and minimize the chances of errors and failure to effectively communicate information [2]. The current study is an evaluation of the efficiency of such structures in improving clinical handoffs and patient safety, discussing their specific frameworks, usage, and influences on the healthcare process because there is very limited literature that directly compares these frameworks.

The SBAR framework can be of great value in high-acuity and emergency situations when communication should be straightforward and unambiguous [3]. SBAR guarantees that relevant information is adequately conveyed among healthcare professionals because it organizes the information in the following approach: situation, background, assessment, and recommendation [4]. SBAR decreases communication disintegration and improves collaboration and patient outcomes through fewer delays in servicing them. The orderly nature of this framework also makes it highly applicable in an environment that demands quick decision-making [5,6]. The importance of nurses' roles and approaches in managing patients with critical conditions is crucial. For instance, Kudu et al., in their study emphasizing the nursing considerations of carotid blowout syndrome, underscore the need for timely, well-coordinated nursing interventions in life-threatening scenarios [7]. 

Conversely, the SOAP framework is based more on the problem-oriented process, having the focus on the formalized logical sequence of subjective patient report to objective clinical finding, assessment, and formulation of the plans of care [8]. It is common in nursing and medical records to keep records of patients well and efficiently organized. The advantage of SOAP is that it methodically records the progress of a patient, making the process of transferring the care between shifts and extended care of patients much easier [9]. Empirical evidence shows that the use of SOAP enhances the clarity of documentation, minimizes omissions, and provides evidence-based decision-making, which helps to achieve better patient safety [10]. 

The PIE framework specifically targets the interventions and results applied by nursing and keeps the documentation in line with the nursing documentation process. Through the monitoring of PIE, it will make sure that nursing practices are always documented and measured in terms of their effectiveness [11]. The model also promotes the continuous review and modification of care plans, thereby generating a dynamic management approach towards a patient [12]. The focus of PIE on real-time documentation and outcomes measurement is of great advantage when frequent monitoring and changes of intervention are required, which is common in long-term or chronic care [13]. 

Although each of the frameworks has its own strengths, their efficacy cannot be experienced unless they are enhanced by effective implementation, training, and interdisciplinary work. Knowledge of the unique uses can assist health facilities in implementing the most appropriate framework for their healthcare requirements. This literature review investigates how SBAR, SOAP, and PIE could aid in enhancing clinical handoffs, focusing on the aspects of their structures, advantages, and disadvantages. This paper will review the current literature on the topic to give an understanding of the best practices in nursing documentation and interprofessional communication to eventually transform healthcare delivery towards a safer and more efficient system.

## Review

Methodology

This systematic review followed the Preferred Reporting Items for Systematic Reviews and Meta-Analyses (PRISMA) guidelines. The research question was formulated using the Population, Intervention, Comparison, and Outcome (PICO) framework [14], which focuses on evaluating the effectiveness of structured nursing documentation frameworks, specifically SBAR, SOAP, and PIE, in enhancing clinical handoffs and improving patient safety outcomes.

Table 1 outlines the PICO framework applied in this review, which focuses on the evaluation of nursing documentation frameworks (SBAR, SOAP, and PIE) in enhancing clinical handoffs and patient safety.

**Table 1:** PICO framework PICO: Population, Intervention, Comparison, and Outcome; SBAR: Situation, Background, Assessment, Recommendation; SOAP: Subjective, Objective, Assessment, Plan; PIE: Problem, Intervention, Evaluation; Mesh: Medical Subject Headings

Concepts	Text words	Controlled vocabulary
Population/Problem	"Nurses", "Registered Nurses", "Clinical Nurses", "Nursing Staff", "Hospital Nurses",	"Nurses" [Mesh], "Nursing Staff" [Mesh], "Hospital Staff" [Mesh]
Intervention	"SBAR Framework", "SOAP Notes", "PIE Documentation", "Structured Documentation Tools", "Handoff Tools", "Communication Frameworks in Nursing"	"SBAR" [Mesh], "SOAP Note" [Mesh], "PIE Format" [Mesh], "Nursing Records" [Mesh], "Communication" [Mesh]
Comparison	None	None
Outcomes	"Communication Effectiveness", "Patient Safety", "Reduction in Errors", "Documentation Accuracy", "Patient Handoff", "Adherence", "Clinical Efficiency"	"Communication" [Mesh], "Patient Safety" [Mesh], "Medical Errors" [Mesh], "Documentation" [Mesh], "Efficiency" [Mesh], "Patient Handoff" [Mesh]

Research Question

What is the effectiveness of nursing documentation frameworks (SBAR, SOAP, and PIE) in enhancing clinical handoffs and patient safety?

Search Strategy

The aforementioned keywords and Medical Subject Headings (Mesh) terms were used with Boolean operators such as AND and OR on three electronic databases (PubMed, Web of Science, and Cochrane Library). The search strings used on each database are given in Table 2.

**Table 2:** Search strings SBAR: Situation, Background, Assessment, Recommendation; SOAP: Subjective, Objective, Assessment, Plan; PIE: Problem, Intervention, Evaluation; TS: topic-specific; TIAB: title and abstract; Mesh: Medical Subject Headings

Database	Search strings
PubMed	("Nurses" [Mesh] OR "Nursing Staff" [Mesh] OR nurses [tiab] OR "registered nurses" [tiab] OR "clinical nurses" [tiab]) AND ("SBAR" [tiab] OR "SBAR communication" [tiab] OR "SOAP note" [tiab] OR "SOAP documentation" [tiab] OR "PIE documentation" [tiab] OR "PIE charting" [tiab] OR "nursing documentation" [Mesh] OR "structured documentation" [tiab]) AND ("Traditional documentation" [tiab] OR "routine charting" [tiab] OR "unstructured notes" [tiab] OR "standard practice" [tiab]) AND ("Patient Safety" [Mesh] OR "Medical Errors" [Mesh] OR "Communication" [Mesh] OR handoff [tiab] OR "nurse satisfaction" [tiab] OR "error reduction" [tiab] OR "clinical handoff" [tiab] OR "documentation accuracy" [tiab])
Web of Science	TS= ("SBAR" OR "SOAP" OR "PIE" OR "Nursing Documentation") AND TS= ("Nurses" OR "Nursing Staff") AND TS= ("Patient Safety" OR "Communication Effectiveness" OR "Clinical Handoff" OR "Error Reduction" OR "Nurse Satisfaction")
Cochrane Library	("Nursing Documentation" OR "SBAR" OR "SOAP" OR "PIE Framework") AND ("Nurses" OR "Healthcare Providers") AND ("Communication in Handoffs" OR "Patient Safety" OR "Error Reduction" OR "Documentation Accuracy")

No restrictions were placed on language during the search, but only studies published in English within the last 20 years, from January 2005 to May 2025, were included during screening. Additionally, manual screening of reference lists from all included articles and relevant reviews was performed to identify any studies missed during the electronic search.

Studies Included

This review included randomized controlled trials, quasi-experimental studies, and mixed-method studies that investigated the use of structured nursing documentation frameworks, specifically SBAR, SOAP, and PIE, in improving clinical handoffs and enhancing patient safety. Eligible studies focused on registered nurses or nursing staff in inpatient, acute care, or hospital-based settings. Studies were included if they evaluated outcomes related to communication effectiveness, accuracy of information transfer, error reduction, and documentation quality during patient handoffs. Interventions had to involve systematic implementation or training in the SBAR, SOAP, or PIE formats. Only studies published in the last 20 years, written in English, involving human participants, and with open-access full-text availability were considered. Key outcomes of interest included communication clarity, reduction in adverse events or medical errors, nurse satisfaction, adherence to the framework, and the overall impact on patient safety and care continuity.

Studies Not Included

The review excluded cohort, case-control, and observational studies, case reports, case series, conference abstracts, editorials, letters, review papers, and meta-analyses. Studies on teenagers, children, and animals were also excluded. Studies that related patient outcomes to the effectiveness of frameworks other than nursing documentation (such as SBAR, SOAP, and PIE), published before January 2015, and those with restricted data access or incomplete analysis were excluded.

Study Selection Process

Initial screening included independent reviewers reading the articles' titles and abstracts. Then, the two independent reviewers conducted a full-text review by comprehensively reading the articles. Regarding reviewers' disagreement, a consensus was developed through the involvement of a third reviewer or through resolution [15].

Methodological Quality Assessment

The quality of the studies included in the research was assessed using the Cochrane Risk of Bias (RoB) 2.0, Risk of Bias in Non-randomized Studies of Interventions (ROBINS-I) tool, and Mixed Methods Appraisal Tool (MMAT). All of these domains were determined to be of low risk, of some concern, and of high risk of bias. Risk of bias was assessed by independent reviewers, and in case of disagreement, they were sorted out mutually, or a third reviewer was consulted for consensus. The evaluation was highly transparent, as it was very consistent and objective due to this strict procedure [16-18]. In order to make the studies more transparent and to provide improved visual interpretation, the authors generated risk of bias traffic lights and summary plots with the robvis tool [19].

Data Extraction and Synthesis

This systematic review conducted the data extraction process with Microsoft Excel 365 (Microsoft Corp., Redmond, WA, USA) [20]. Independent reviewers extracted data in parallel after completing calibration exercises on three randomly selected studies to ensure consistency in interpretation and minimize variability. Disagreements were resolved through discussion, with arbitration by a third reviewer when necessary. Extracted data were organized for screening and reviewer assignment. Extracted data included author and year, along with the study design (e.g., randomized controlled trials, quasi-experimental studies, or mixed-method studies) and sample size, focusing exclusively on nurses. The specific documentation frameworks assessed, such as SBAR, SOAP, and PIE, were recorded. Details regarding implementation strategies, including training programs, duration, and integration into clinical workflow, were noted. Handoff outcomes such as communication clarity, completeness of information, and handover efficiency were evaluated. Patient safety indicators, including error rates, adverse event reporting, and continuity of care, were extracted where available. Evaluation metrics involved both objective measures (e.g., number of reported incidents, documentation time) and subjective assessments (e.g., nurse satisfaction, perceived usability). Key findings highlighted the relative effectiveness of each framework in improving communication and care quality. Additionally, studies were analyzed for reported barriers, such as resistance to change, time constraints, lack of training, and limitations, including small sample sizes, short follow-up periods, and limited generalizability across settings.

The synthesis process followed four clearly defined steps to ensure a rigorous and transparent analysis of the effectiveness of nursing documentation frameworks. (1) In the initial coding, two reviewers independently reviewed and coded both quantitative and qualitative data (e.g., handoff quality, communication accuracy, error reduction, and nurse satisfaction) into preliminary outcome categories such as clinical communication effectiveness, patient safety impact, and user adherence. (2) In the theme development, these initial codes were organized into broader thematic domains, including communication outcomes (e.g., clarity, completeness, standardization), safety outcomes (e.g., reduction in medical errors, continuity of care), and usability outcomes (e.g., nurse satisfaction, framework adherence). (3) In the theme refinement and validation, the reviewers jointly examined the thematic structure, resolved any discrepancies through discussion, and refined the categories to reduce overlap and ensure conceptual clarity across studies. (4) In the final synthesis, the finalized themes were synthesized narratively and presented alongside comparative tables to facilitate cross-study interpretation and highlight recurring patterns. This thematic synthesis approach enabled the meaningful integration of findings from heterogeneous study designs and outcome measures, supporting clinically relevant conclusions regarding the implementation and effectiveness of SBAR, SOAP, and PIE frameworks in nursing practice.

Ethical Consideration

**Figure 1:**
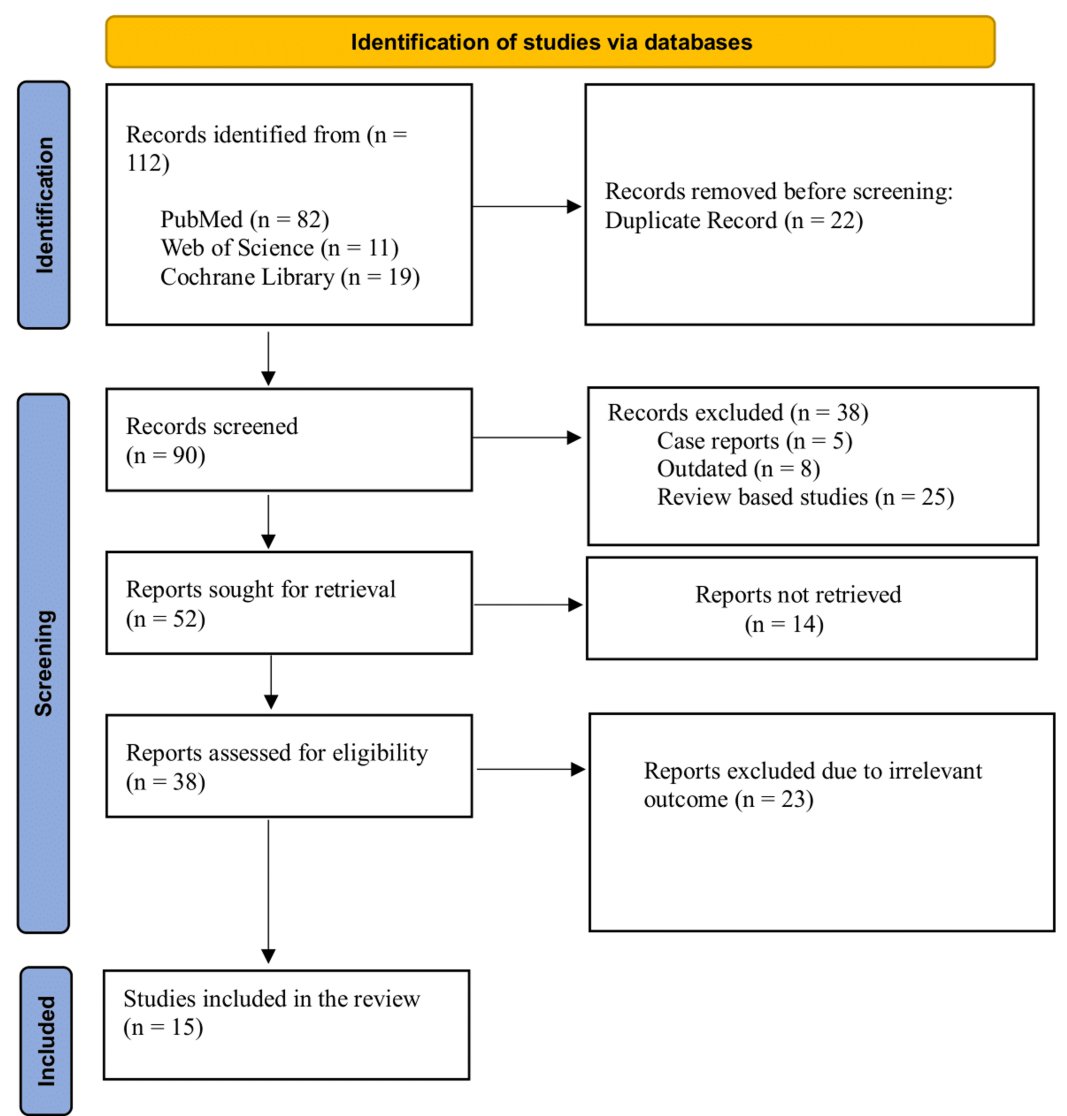
PRISMA flowchart PRISMA: Preferred Reporting Items for Systematic Reviews and Meta-Analyses

As this study is a systematic review of previously published literature and involves no direct human or animal subjects, formal ethical approval was not required. No personal or identifiable data were collected, and issues of confidentiality or anonymity do not apply. The review was conducted with transparency and rigor, adhering to PRISMA 2020 guidelines to ensure reproducibility [21]. The literature search process was presented in Figure 1 as a PRISMA flowchart.

Results

A total of 112 articles were initially identified through database searches (PubMed = 82; Web of Science = 11; Cochrane Library = 19). After removing 22 duplicates, 90 articles were screened based on titles and abstracts. A total of 38 were excluded from the review, including five case reports, eight outdated studies, and 25 review-based studies. A total of 52 reports were sought for retrieval; out of these, 14 studies could not be included due to the unavailability of full-text articles. The remaining 38 reports were assessed for full-text eligibility. The twenty-three articles of irrelevant outcomes were excluded during the screen phase 2. Ultimately, 15 studies were included in the review for quality assessment.

Risk of Bias Assessment

**Figure 2:**
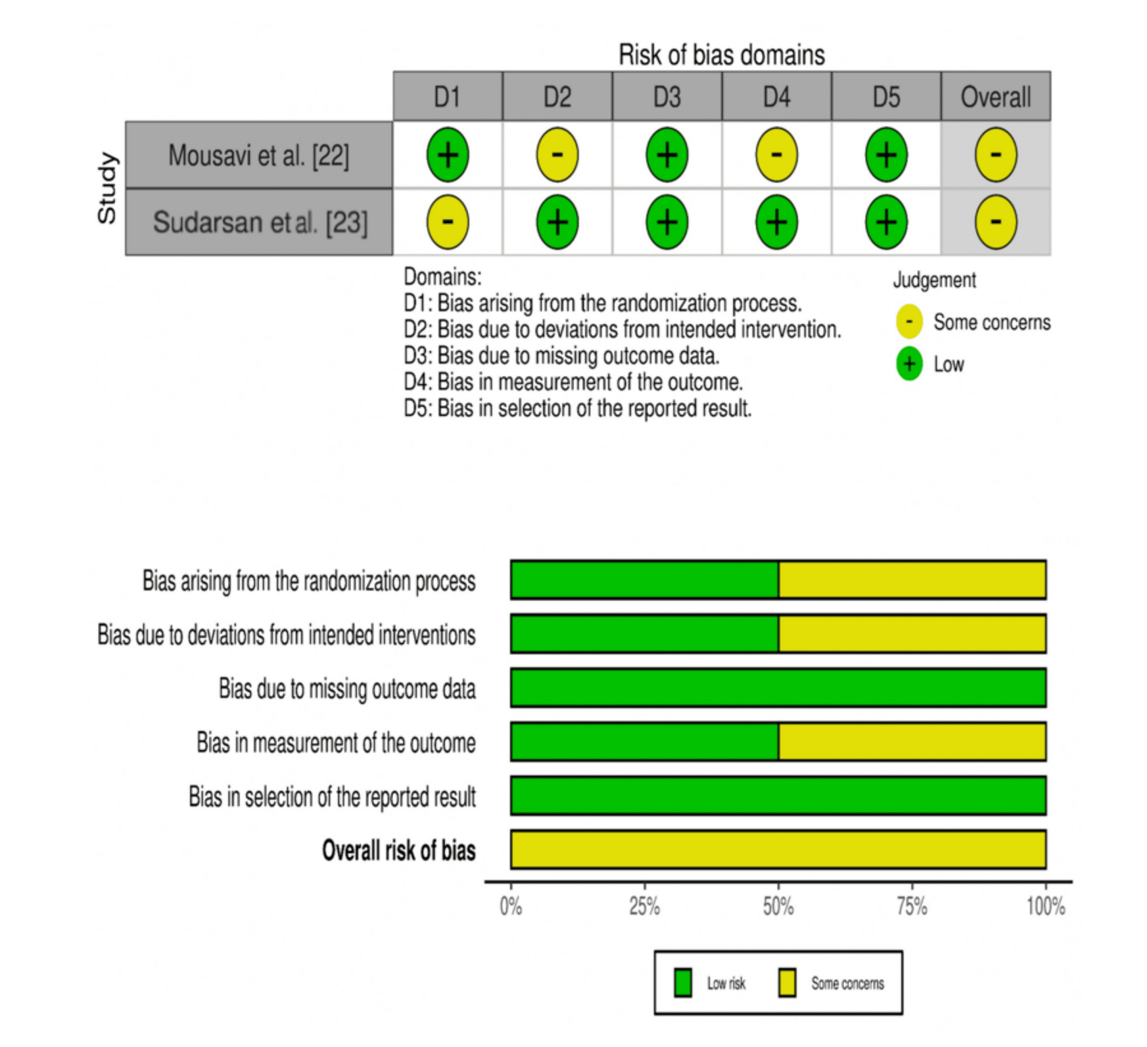
Traffic light and summary plots using the Cochrane Risk of Bias (RoB) 2.0

Figure 2 illustrates the Cochrane RoB 2.0 tool, which was used to assess the methodological quality of the included randomized studies across five domains. Mousavi et al. showed low risk in most domains, with some concerns in deviations from intended interventions and outcome measurement [22]. Sudarsan et al. had low risk across four domains, with some concerns related to the randomization process [23]. Overall, both studies were rated as having "some concerns" regarding risk of bias.

**Figure 3:**
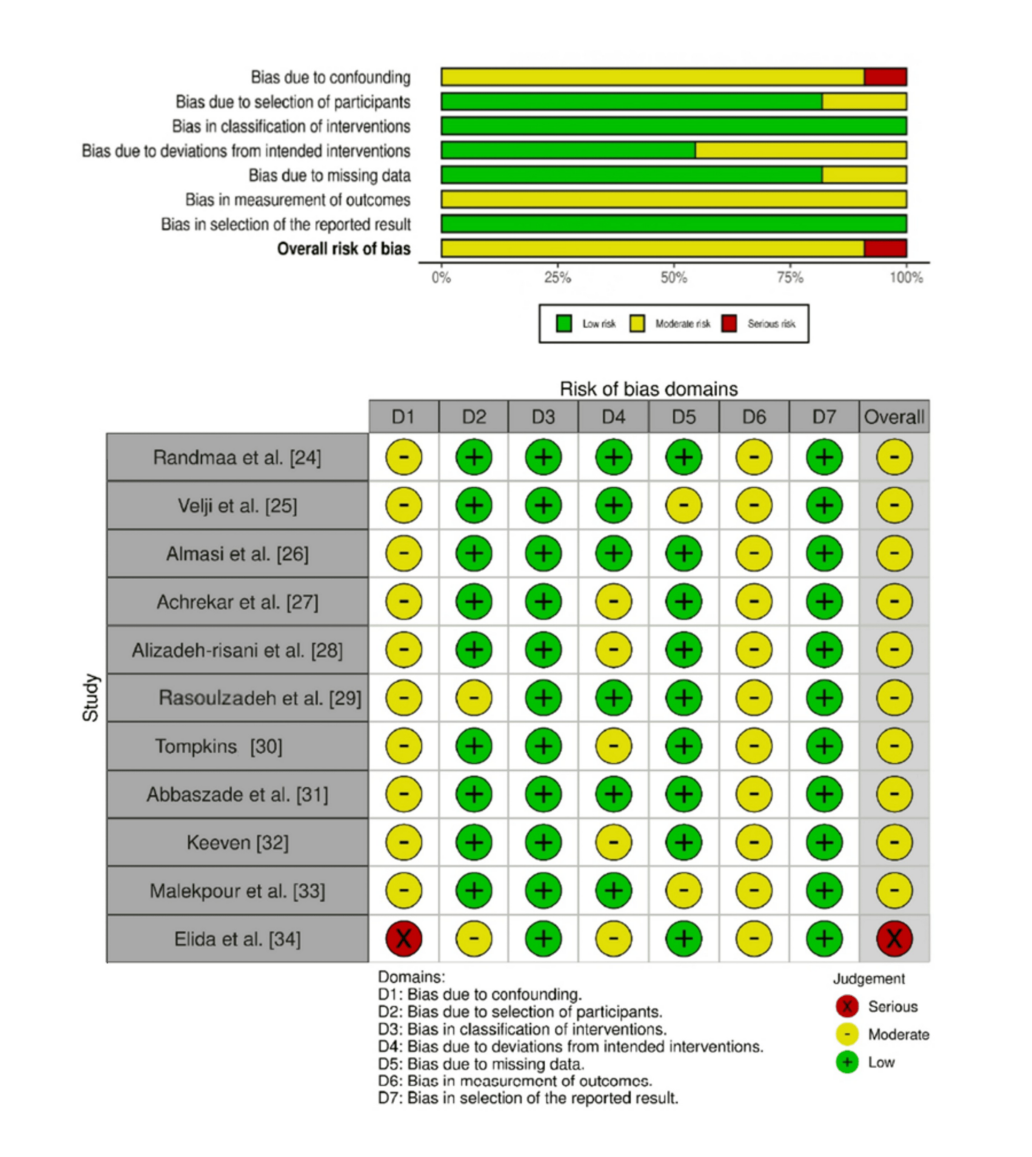
Traffic light and summary plots of ROBINS-I ROBINS-I: Risk of Bias in Non-randomized Studies of Interventions

The ROBINS-I tool was used to assess ROB in seven domains of the studies, which were selected as non-randomized. The most dominant bias was confounding; however, most of the studies had a moderate risk of bias, like the studies by Randmaa et al., Velji et al., and Almasi et al. [24-26]. In all studies, risks of bias in participant selection, the categorization of interventions, and reporting were mostly reported to be low, implying that those areas were designed and conducted appositely [27-33]. In almost every study, moderate risk in measurement of the outcomes was noted, which indicated the possible weaknesses in outcome assessment methodology. Some studies, including those by Elida et al., had a high degree of bias, with confounding as one of the main sources along with the selection of participants [34]. The amount of missing data or the failures to take the intended interventions were rated as low or moderate across studies. On the whole, most of the studies fell into the category of moderate overall risk of bias and thus a satisfactory inclusion and interpretation of results in this review (Figure 3).

Table 3 presents the MMAT for mixed-method studies included in the systematic review. MMAT was applied to evaluate the quality of methods of the included studies. The scores given to both Skingley et al. and Groves et al. were 4/5, which indicates a good quality with restrictions [35,36]. Low biases in selection and attrition were observed in both studies, but their biases on detection and on other biases were moderate. All in all, every study was ranked to have a moderate risk of bias.

**Table 3:** MMAT score MMAT: Mixed Methods Appraisal Tool

Study	MMAT score	Selection bias	Performance bias	Detection bias	Attrition bias	Reporting bias	Other bias	Overall risk of bias
Skingley et al. [35]	(4/5)	Low	Moderate	Moderate	Low	Low	Moderate	Moderate
Groves et al. [36]	(4/5)	Low	Low	Moderate	Low	Low	Moderate	Moderate

Characteristics and Findings of the Studies Included in the Review

The studies evaluating structured communication frameworks SBAR, SOAP, and PIE employed various designs across diverse clinical and educational settings. Most used quasi-experimental pre-post intervention designs, such as Randmaa et al. in an anesthetic clinic (n = 169), Velji et al. in a stroke rehabilitation unit (n = 43), and Achrekar et al. in oncology units (n = 20) [24,25,27]. Mousavi et al. conducted a randomized controlled trial with 120 nurses to assess SOAPIE (Subjective, Objective, Assessment, Plan, Intervention, Evaluation) documentation, while Sudarsan et al. used a randomized crossover design to compare SOAP and Advanced SOAP (A-SOAP) formats among 34 pharmacy students [22,23]. Alizadeh-risani et al. compared SBAR with a modified handover model in an emergency department involving 31 nurses [28]. PIE framework studies included Almasi et al. and Rasoulzadeh et al., who evaluated training outcomes among nursing students (n = 28 and n = 30, respectively), and Skingley et al., who conducted a qualitative study across 10 hospital wards in the UK using normalization process theory [26,29,35]. Tompkins analyzed 110 SBAR forms in a VA hospital using a revised tool, while Abbaszade et al. assessed SBAR's impact on patient care quality in a coronary care unit (n = 144) [30,31]. Groves et al. used a simulation-based design with 20 nurses to test safety priming during handoffs [36]. In a pediatric intensive care unit (PICU), Keeven employed a mixed-method approach across 4,233 handoffs to evaluate a standardized tool and checklist [32]. Elida et al. explored SOAP documentation among 53 nurses in Indonesia, and Malekpour et al. studied SOAP training effects on 832 medical records completed by 10 surgical assistants [33,34]. These studies collectively represent a wide range of methodologies and healthcare contexts, offering versatile insights into the implementation and effectiveness of structured communication tools. Table 4 presents the characteristics of the included studies.

**Table 4:** Characteristics of the included studies SBAR: Situation, Background, Assessment, Recommendation; AHRQ: Agency for Healthcare Research and Quality; ICU: intensive care unit, SAQ: Safety Attitudes Questionnaire; CPRS: Clinical Patient Reported Satisfaction; SIM: self-instructional module; SOAPIE: Subjective, Objective, Assessment, Plan, Intervention, Evaluation; SOAP: Subjective, Objective, Assessment, Plan; NPHQ: Nurse Perception of Handover Questionnaire; HQRT: Handover Quality Rating Tool; QUALPACS: Quality of Care and Patient Safety; PIE: Problem, Intervention, Evaluation; PIE Programme: Problem, Intervention, Evaluation Programme; NPT: normalization process theory; PET: Practice, Evidence, Translation; EHR: electronic health record; SEMS: safety event monitoring system; McNemar: a statistical test used to evaluate paired nominal data; A-SOAP: Advanced SOAP; T1, T2: time 1 and time 2 (used in various survey stages); RNs: registered nurses; EBP: evidence-based practice; PIV: peripheral intravenous; ANCOVA: analysis of covariance; SAPS: Safety Action Performance Scale

Author, year	Study design, sample size	Frameworks assessed	Implementation	Handoff outcomes	Patient safety	Evaluation metrics	Key findings	Barriers	Limitations
Randmaa et al. [24]	Quasi-experimental pre-post intervention design group: 100 staff (baseline: 139; follow-up: 100). Comparison group: 69 staff (baseline: 91; follow-up: 69)	SBAR communication tool	Modified SBAR pocket cards, in-house training (2.5 hours), role-playing, informational materials, and 7 months of monitored implementation with structured observations (168 handovers). Manipulation checks via monthly randomized telephone interviews	Significant improvement in between-group communication accuracy (p = 0.001). No significant change in within-group communication openness or timeliness	Reduction in incident reports related to communication errors. Improved the perception of safety climate among staff	Questionnaires: ICU Nurse Physician Questionnaire, SAQ, Spreitzer's Empowerment Scale. Incident reports: Analyzed for communication errors pre- and post-intervention. Statistical tests: Wilcoxon signed-rank test, Mann-Whitney U test, χ² test, and Fisher's exact test	SBAR improved between-group communication accuracy and safety climate. Significant decrease in incident reports due to communication errors. No significant change in psychological empowerment	Selection bias: Non-randomized design. Baseline differences: The comparison group had higher baseline scores in some factors (e.g., teamwork climate, safety climate). Dropout differences: Dropouts had fewer years of experience and different scores on some factors	Quasi-experimental design: Lack of randomization. Potential diffusion: Intervention effects may have spread to the comparison group. Baseline differences: Could affect the interpretation of results. Self-reported data: Subject to bias
Velji et al. [25]	Quasi-experimental pre-post intervention design: 43 staff (32 at baseline, 27 at follow-up)	SBAR communication tool	Phase 1: SBAR adapted via focus groups with staff, patients, and families. Phase 2: SBAR implemented over 6 months via workshops (4 hours total) with role-playing and case examples. Phase 3: Evaluation using surveys, patient feedback, and safety reports	Staff perceptions: Significant improvements in 5/12 safety culture domains (p < 0.05), including communication openness and feedback about errors. Team communication: SBAR used for urgent/nonurgent issues (e.g., discharge planning, debriefs)	Safety culture: The stroke unit outperformed the hospital in 7/12 domains post-intervention (e.g., organizational learning, feedback). Incident reporting: Increased trends in reporting (attributed to broader hospital initiatives)	Quantitative: AHRQ survey (5-point Likert scale), CPRS (patient satisfaction), incident reports. Qualitative: Focus group feedback guiding SBAR adaptation. Statistical tests: Critical ratio tests, unpaired t-tests, and the 5% "rule of thumb" for meaningful differences	SBAR improved staff perceptions of communication and safety culture. Marginal, non-significant improvements in patient satisfaction (ceiling effect). Increased incident reporting (though small sample size limited significance). SBAR expanded beyond acute care to rehabilitation (interprofessional use)	Small sample size: Limited statistical power. Ceiling effect: High baseline scores in patient satisfaction. Confounding factors: Hospital-wide safety initiatives may have influenced results	Non-randomized design: Single-unit intervention. Short follow-up: 6 months post-implementation. Low response rates: 27% (T1), 22% (T2) hospital-wide. Attribution challenges: Improvements may reflect broader organizational changes
Achrekar et al. [27]	Quasi-experimental pre-post intervention design: 20 nurses (first audit), 19 nurses (second audit), 17 nurses (opinion survey)	SBAR	Introduced SBAR via a SIM; form disseminated for clinical use during shift handovers. Audits conducted in the first week (A1) and the 16th week (A2)	Significant improvement in overall SBAR documentation scores (p = 0.043). Highest improvement in "Situation" domain (p = 0.045). 76% of nurses found SBAR useful, but 24% reported it as time-consuming	Improved communication during transitions of care. Gaps identified in documenting allergies, comorbidities, and plan of care (70% compliance). 63% of nurses believed SBAR would improve patient safety	Compliance scores for SBAR domains (Situation, Background, Assessment, Recommendation). Nurse opinions via Likert scale surveys. Statistical analysis (Wilcoxon signed-rank test, McNemar test)	SBAR improved structured communication during handovers. Graduate nurses performed better than diploma nurses (p = 0.019). Challenges: Time constraints, inconsistent documentation of critical details (e.g., allergies, comorbidities)	Time-consuming for some nurses (24%). Lack of clarity in documenting certain domains (e.g., "Recommendation"). Low patient involvement (only 53% of nurses deemed it necessary)	Small sample size (not generalizable). Self-reported data may lack accuracy. Patient outcomes (e.g., length of stay) were not evaluated. No content analysis of all SBAR forms
Mousavi et al. [22]	Randomized controlled trial: 120 nurses (60 intervention, 60 control)	SOAPIE	3-week SOAPIE training workshop for the intervention group. No intervention for the control group. Data collected before and one month post-intervention	Significant improvement in nursing documentation quality in the intervention group (p = 0.001). No significant change in the control group (p = 0.23)	Improved documentation accuracy and completeness. Reduced errors in nursing notes, enhancing care continuity	Nursing documentation checklist (11 principles scored 0-2). Three quality levels: Poor (0-33%), moderate (34-66%), and favorable (67-100%). Statistical tests (chi-squared test, independent t-test, paired t-test)	Post-intervention, the intervention group's documentation quality improved from 46.66 ± 14.45 to 91.53 ± 5.98 (p = 0.001). SOAPIE method significantly enhanced the clarity, structure, and completeness of nursing notes	Heavy workload and nurse fatigue (especially during COVID-19). Time constraints for documentation. Lack of prior training in structured documentation methods	Small sample size (limited generalizability). Potential bias due to self-reported checklist data. Short follow-up period (1 month). No evaluation of long-term patient outcomes
Sudarsan et al. [23]	Randomized controlled trial: 34 postgraduate students	SOAP 1: Basic SOAP. A-SOAP: Advanced SOAP (enhanced with pharmacy-specific components)	Randomized administration of SOAP 1 and A-SOAP for the same case scenario. Feedback collected via Likert scale questionnaire. Blinded evaluation using a rubric grading tool	Improved interprofessional communication through structured documentation	Reduced misinterpretation of patient data. Enhanced the identification of drug-related problems (e.g., interactions, dosing errors)	Scores: Grading rubric (0-100 points) for intervention quality. Time: Documentation time. Feedback: Likert-scale responses (relevance, usability)	A-SOAP scores (57.94 ± 15.86) significantly higher than SOAP 1 (14.49 ± 12.95; p < 0.001). Improved problem identification and prioritization. 76% of students reported better intervention identification with A-SOAP	Time-consuming (average 45 minutes for A-SOAP). Varied understanding among students (fourth vs. fifth vs. sixth years)	Small sample size (n = 34). Single-case scenario (generalizability untested). No long-term follow-up
Alizadeh-risani et al. [28]	Quasi-experimental (pre-post-intervention): 31 nurses (ED setting)	SBAR vs. modified handover model (written checklist + bedside handover)	SBAR: Routine oral handover. Modified model: 1-hour theory session + 3 hands-on bedside training sessions; implemented for 1 month (~350 handovers)	Modified model outperformed SBAR in information transfer (p < 0.001; Cohen's d = 1.56); shared understanding (p < 0.001; d = 1.09); working atmosphere (p = 0.004; d = 0.56); overall handover quality (p < 0.001; d = 1.29); and nurse perception (p < 0.001; d = 1.51)	Improved documentation completeness (e.g., allergies, vital signs). Reduced omissions in care plans. Enhanced patient/family involvement in handovers	NPHQ: 22-item Likert scale (Cronbach's α = 0.93). HQRT: 16-item Likert scale (α = 0.96). Statistical tests: Paired t-tests, Cohen's d effect size, ANCOVA	Modified model improved handover quality by 8.09 points (vs. SBAR). Highest gains in information transfer (e.g., use of charts, medication details). 79% of nurses preferred the modified model for clarity and structure	Nurse resistance to change (resolved via training/support). Time constraints for training/implementation. Complex cases (e.g., critical care) required adaptations	Small sample (single ED; limits generalizability). Short follow-up (1 month). No long-term patient safety outcomes (e.g., error rates). Potential bias from self-reported data
Skingley et al. [35]	Mixed-method (10 wards across 5 NHS hospital trusts in the UK). Qualitative focus with NPT as the analytical framework. 10 hospital wards (2 fully implemented PIE, 2 partially implemented, 6 non-implementers). Staff included nurses, therapists, and healthcare assistants	PIE Programme: Cycles of observation, reflection, action planning, and review. NPT: Coherence, cognitive participation, collective action, and reflexive monitoring	Phases: Exploration, program installation, initial implementation, full adoption, sustainability. Activities: Staff training workshops, real-time observations, collaborative action plans. Duration: 18 months for full implementers	Improved interprofessional collaboration and communication in full-implementer wards (e.g., shared goals for person-centered care)	Reduced isolation and neglect (e.g., staggered staff breaks, improved mealtime interactions). Enhanced monitoring of environmental factors (noise, temperature)	Qualitative: Interviews, observation notes, workshop feedback. NPT mechanisms: Coherence, participation, collective action, and reflexive monitoring. Implementation stages: Fixsen et al.'s (2005) stages (exploration to sustainability)	Full implementers (2 wards): Sustained practice changes (e.g., social mealtimes, music therapy). Partial implementers (2 wards): Initial enthusiasm stalled due to resource constraints. Non-implementers (6 wards): Failed due to organizational turbulence (e.g., ward closures, staff shortages). Critical success factors: Leadership, facilitation, organizational stability, and resource adequacy	Resource constraints: Staff shortages, time limitations. Organizational instability: Trust restructuring, ward closures. Leadership gaps: Lack of sustained external facilitation	Small scale: Only 2 wards fully implemented PIE. Time-bound: 18-month follow-up; sustainability challenges noted. Contextual bias: All wards in the UK NHS; limited generalizability. No patient outcomes: Focused on process, not clinical results
Almasi et al. [26]	Quasi-experimental pre-post-intervention design (single group): 28 nursing students	PIE vs. traditional/narrative documentation	Pre-intervention: Students wrote 2 traditional reports. Intervention: 1-week PIE training workshop (theory + practice). Post-intervention: Students wrote 2 PIE reports	Significant improvement in documentation quality (p < 0.001). Higher scores in structure (16.67 → 25.46) and content (14.39 → 24.71). 53.6% of post-intervention reports were rated "completely desirable" (vs. 7.1% pre-intervention)	Improved accuracy in documenting nursing diagnoses, interventions, and outcomes. Reduced omissions in patient care records	21-item checklist: Scored 0-3 (structure: 10 items; content: 11 items). Quality levels: Undesirable (<25%), somewhat desirable (25-50%), completely desirable (>50%). Statistical tests: paired t-tests	The PIE method significantly improved documentation quality (mean score: 31.03 → 50.17). Enhanced adherence to the nursing process (problem identification, interventions, evaluation). Students preferred PIE for its structured approach	Prior lack of PIE knowledge among students. Time constraints for training. Potential peer influence during study (uncontrolled sharing of PIE knowledge)	Small sample size (single institution). Short follow-up (no long-term evaluation). Potential bias from uncontrolled peer interaction. No comparison with other problem-oriented methods (e.g., SOAP)
Rasoulzadeh et al. [29]	Quasi-experimental single group, pre- and post-test: 30 nursing students	PIE	Training nursing students in the PIE method for recording patient care in surgical and internal departments	Improved record quality, structured documentation, and better communication among care providers	Enhanced accuracy and completeness of records, leading to better patient care	Checklist for nurse record quality (21 items scored 0-4), paired t-test, Mann-Whitney U test	PIE training significantly increased record quality (mean score: 49.93% to 77.45%; p < 0.001)	Lack of awareness, poor management, and inadequate training in traditional methods	Small sample size, single-group design, no control group, limited to one geographic area
Tompkins [30]	Quasi-experimental pre-post-intervention design of 110 SBAR forms (pre- and post-implementation)	SBAR: Revised tool with checklist. Johns Hopkins Nursing EBP Model PET. Kotter's 8-Step Change Model	Intervention: Revised SBAR tool with expanded documentation space and safety checklist. Training: Staff education via workshops and reminders. Duration: 8 weeks. Change management: Kotter's model (e.g., creating urgency, stakeholder engagement)	100% communication effectiveness (vs. 77.14% pre-implementation). 96.87% accuracy in tracking invasive devices (e.g., central lines, nasogastric tubes). 90.91% compliance with restraint documentation (vs. 6.06% pre-implementation)	Reduced errors in PIV dressing changes, restraint orders, and medication administration. Improved the continuity of care during transitions	Quantitative: Chi-squared tests for SBAR completion/helpfulness. Qualitative: Staff surveys on usability (Likert scale). Audits: Tracking compliance with key safety metrics (e.g., IV dressings, restraints)	Statistically significant improvements (p < 0.001) in documentation accuracy and completeness. Staff reported time savings and ease of use with the revised SBAR. Enhanced interdisciplinary collaboration during handoffs	Staffing shortages and float nurses unfamiliar with the tool. Double charting due to parallel documentation systems. Resource limitations (e.g., lack of pencils/erasers)	Single-site study (limited generalizability). Short duration (8 weeks). Sample bias (VA hospital setting). Reliance on self-reported audits
Abbaszade et al. [31]	Quasi-experimental (pre-post-intervention): 144 patients (72 pre-intervention, 72 post-intervention)	SBAR	Pre-intervention: Traditional verbal bedside handoff. Intervention: Nurses trained in SBAR via 5 one-hour sessions; SBAR checklists provided. Post-intervention: SBAR implemented for 1 week under supervision	Significant improvement in QUALPACS scores: Psychosocial: 55.34 → 67.70 (p < 0.001). Physical: 48.86 → 60.18 (p < 0.001). Communication: 23.86 → 30.09 (p < 0.001)	Enhanced the continuity of care. Reduced miscommunication errors. Improved nurse-patient rapport and trust	QUALPACS (65-item scale): Psychosocial (28 items), physical (24 items), and communication (13 items). Scores: 0-3 (undesirable to ideal). Statistical tests: ANCOVA (adjusted for covariates)	SBAR improved all dimensions of nursing care quality. Highest impact on psychosocial care (e.g., reduced patient anxiety). Better nurse-patient and interprofessional communication	No random allocation of hospitals/patients. Varied nurse-to-patient ratios and organizational styles between hospitals	Non-randomized design (potential confounding variables). Short implementation period (1 week). Limited to CCU settings (generalizability uncertain)
Groves et al. [36]	Quasi-experimental pre-post-intervention design: 20 RNs randomized into intervention (n = 10) and control (n = 10) groups	Structuration theory of safety culture: Safety priming framework (communication → goal activation → behavior)	Safety priming intervention embedded in shift-to-shift handoff communication (e.g., explicit safety cues like "high risk for falls")	The intervention group completed slightly more safety actions (60.5% vs. 57.9%), but the difference was non-significant	Focus on safety-oriented behaviors (e.g., fall prevention, infection control) in response to embedded risks	SAPS: 43-item and reduced 23-item versions; stimulated recall interviews	Small, non-significant increase in safety actions with priming; routinized behaviors less affected by priming	Competing clinical demands; the complexity of real-world nursing not fully captured in simulation	Small sample size; simulation may lack ecological validity; priming limited to handoff context
Keeven [32]	Mixed-method: 4,233 handoffs (788 using the new tool, 18.62% compliance)	Standardized nursing handoff tool (two components: handoff communication tool + patient safety checklist)	Reintroduced the standardized handoff tool in the PICU, included staff education and monitoring, and conducted over 12 weeks	60% reduction in reported patient harm events (not statistically significant; p = 0.614). Low tool utilization (18.62%). No significant change in SEMS-reported harm events (p = 0.489)	46 handoff-related events reported (3 resulting in harm). Aimed to reduce safety events by 10% (outcome not statistically significant)	Compliance with handoff tool (retrospective chart review). SEMS-reported patient harm events. Fisher's exact test for statistical analysis	Reduction in harm events (though not statistically significant). Low adoption due to tool design and staff resistance. Highlighted the need for EHR integration and workflow adjustments	Staff resistance to change. Spatial constraints in PICU. Perceived inefficiency of tool organization. Lack of EHR prompts for documentation	Small sample size for harm events. Short implementation period (12 weeks). Low compliance (18.62%). No randomization or control group
Elida et al. [34]	Quasi-experimental pre-post-intervention design: 53 nurses at Hospital X, Bogor Regency	Traditional SOAP documentation	Training intervention on SOAP documentation, followed by simulations and assistance for 3 days, with evaluation after 3 weeks	Improved completeness of SOAP documentation post-training (increase from 28.3% to 45.2%)	Better documentation reduces errors and enhances the continuity of care during handovers	Pre-test-post-test knowledge scores. Completeness of SOAP documentation (complete/incomplete). Wilcoxon test (knowledge differences). Chi-squared test (documentation completeness)	Significant increase in nurses' knowledge post-training (p = 0.000). 16.9% improvement in SOAP documentation completeness. No significant effect of knowledge/motivation on documentation completeness (p > 0.05). High baseline motivation (86.8%) but did not correlate with documentation outcomes	Lack of standardized abbreviation guides. Rushed documentation habits. Low prioritization of documentation over direct care. Inconsistent supervision	No control group (pre-experimental design). The motivation questionnaire not specific to SOAP documentation. No multivariate analysis to identify confounding factors. Small sample size (53 nurses)
Malekpour et al. [33]	Quasi-experimental: 10 general surgery assistants, 832 medical records (412 pre-intervention, 420 post-intervention)	SOAP method for progress note documentation	Blended learning: Peer-to-peer problem-solving + email-based education on SOAP standards	Improved accuracy and completeness of progress notes in medical records	Enhanced documentation quality supports the continuity of care, legal protection, and service evaluation	Compliance with SOAP standards (pre-/post-intervention). Quantitative (paired t-test) and qualitative (McNemar test) analysis	Significant improvement in documentation of patient status, care measures, and treatment plans (p = 0.005). Increased adherence to physician-related data (name, time, signature) (p = 0.002-0.005). No significant change in chief physician confirmation (p = 0.335)	Lack of prior standardized training. Time constraints for documentation. Variability in assistant experience levels	Small sample size (10 assistants). No control group. Focus limited to general surgery assistants

Impact on Patient Safety

Error reduction: SBAR, SOAP, and PIE structures of communication have been useful in curbing clinical errors because they provide a clear, standardized, and concise flow of information between health practitioners. It was shown that the use of SBAR led to a significant reduction in reported incidents concerning the communication mistakes in an anesthetic clinic (p = 0.001), suggesting increased patient safety as the result of more accurate and clear information [24]. In the same way, Tompkins also found a steep spike in the measure of effectiveness of communications, from 77.14% to 100%, when staff were trained to use an updated SBAR tool that included safety checklists and extra documentation space [30]. This resulted in significant decreases in inaccurate intravenous dressing changes, restraint orders, and medication administration errors. These findings demonstrate that well-designed tools minimize preventable adverse events through less miscommunication, particularly on risky handoffs of care.

Clinical handoffs: The successful clinical handoffs are necessary to ensure the continuity of care, and well-organized documentation frameworks contribute to a high quality and consistency of the handoffs. Alizadeh-risani et al. took standard SBAR oral handoffs and compared them with a modified handover model involving bedside communication and written checklists [28]. The more inclusive method translated into much-improved results relative to information transfer (p < 0.001), shared understanding (p < 0.001), and comprehensive handover (p < 0.001) and suggests safer and more comprehensive handoffs. Similarly, Abbaszade et al. demonstrated the additional benefit of communicative continuity of nursing care because of the improved communication through SBAR use in their sample, which was mirrored in higher Quality of Care and Patient Safety (QUALPACS) scores in the following areas: psychosocial (p < 0.001), physical (p < 0.001), and communication (p < 0.001) [31]. These results reiterate the importance of clear, well-organized handoffs supported by such frameworks as SBAR, SOAP, and PIE, as they directly support better patient conditions by decreasing the interruptions and lapses of continuity.

Implementation and Training

Training programs: Well-developed training programs for healthcare professionals are of high relevance to the successful implementation of structured communication frameworks, like SBAR, SOAP, and PIE. Even the most useful tools can go awry unless they are properly trained to bring a continuous positive outcome in practical application. Various pieces of research indicate the good effect of specific education. Mousavi et al. have performed a structured three-week training program covering SOAPIE documentation for nurses. The quality of documentation improved statistically (p = 0.001), and the scores increased, showing better clarity, structure, and completeness of nursing records, after this intervention went up (from 46.66 + 14.45 to 91.53 + 5.98) [22]. Equally, Almasi et al. documented that the nursing students have considerably improved their documentation scores following a one-week training program on PIE, where the post-intervention mean score increased by 20 points to reach 50.17 (p < 0.001) [26]. These findings make their point of how powerful structured training can be by means of workshops, simulation, or blended learning to standardize and ensure the valid application of documentation techniques.

Adaptation and adherence: Besides their initial institution, long-term success rates of structured communication frameworks depend on their respective suitability to particular clinical settings and enforcement on personnel. Velji et al. proved the advantages of implementing SBAR adjustment with the help of the stakeholder input by involving the staff, patients, and families. Such an inclusive strategy delivered enhancements in a variety of dimensions of safety culture, including but not confined to openness of communications along with feedback on mistakes [25]. Nonetheless, it might be complicated to thoroughly integrate such frameworks. According to Skingley et al., the implementation of the PIE model was successful in only two of 10 wards during the execution of a larger-scale initiative that aims to use person-centered care [35]. The other wards were partially introduced or did not launch at all because of the obstacles in terms of the shortage of personnel, unstable organization, and unsupportive leadership. These contrasting results underline that despite a promising prospect of using the structured communication tools, their effective implementation and long-term sustainability strongly depend on the organizational environment, resource novelty, and commitment to change.

Technological Integration

Electronic health records (EHRs):** **The structured communication frameworks such as SBAR, which are supported as part of EHRs, improve the quality of documentation by increasing the consistency thereof and also facilitating timely access to vital patient information. Tompkins provided evidence that the redesign of the SBAR format with an increase in space and an addition of safety checklists in it, as well as digital documentation integration, produced meaningful differences in terms of accuracy and efficiency [30]. Employees told of less difficulty and less time, and compliance scores, such as restraint documentation kept track, showed a dramatic increase in compliance from 6.06 to 90.91, and tracking of invasive devices showed 96.87% accuracy. Nevertheless, effective digital integration cannot be considered a sure thing. Keeven has found that the average handoff tool in a PICU attained only 18.62% compliance because of several problems, such as inefficient EHR integration, the absence of system prompts, and general inefficiencies in tool design [32]. These mixed results serve to emphasize the notion that to benefit EHR integration, digital tool design should match clinical practice and be intuitive and usable; otherwise, its adoption will be poor, and its effect on patient safety will be minimal.

Digital tools:** **Besides the EHR systems, an assortment of stand-alone digital solutions exists, including templates, mobile applications, and self-instructional modules (SIM), which have been used to facilitate structured communication in healthcare. Such tools are important in enhancing standardization and documentation. As an example, Achrekar et al. implemented a SIM and issued standardized SBAR forms of clinical handovers. As a result of this strategy, the level of SBAR documentation completeness increased by a large margin, with the most significant increment recorded under the Situation domain (p = 0.045) [27]. Similarly, Tompkins documented that their digital version of the SBAR, with in-built safety reminders, led to an increased rate of documentation compliance and was rated highly by staff, who claimed an improvement in usability and efficiencies of workflow [30]. Such results suggest that carefully developed digital technologies may be effective and convenient tools to enhance the precision of communication and minimize variation in the clinical recording process since they become well integrated into the lives of healthcare providers.

Barriers to Implementation and Organizational Challenges

Organizational-level issues usually impede the introduction of structured communication models such as SBAR, SOAP, and PIE. These are inadequate training resources, personnel constraints, executive withdrawal, and time constraints that all may interfere with regular utilization. According to Skingley et al., who examined the 10 hospital wards in their research, these were exactly the problems that explained the lack of success of the PIE model. Two wards were able to fully utilize PIE, whereas others were not successful because of inadequate human resources, external facilitation, and organizational restructuring [35]. Alizadeh-risani et al. stated the same, where despite the value nurses attached to the new modified handover model, real-life factors like time and challenges in the cases of critical care were common obstacles to its complete implementation [28]. These results indicate that the effective assimilation of communication technologies is not just about personnel training but also about proper organizational decision-making and allocation of resources, as well as ensuring that strategies of implementation conform to the circumstances of the clinical workload.

Resistance to Change

A second barrier that is certainly prevalent in the proliferation of standardized communication tools is that of staff resistance, particularly by nurses. The fear of using these tools is also commonplace, as they are viewed as cumbersome or even time-consuming, and resistance to changing existing methods of documentation is also a possible cause. Respondents in the survey by Achrekar et al. stated that adopting SBAR was time-consuming since 24% of the nurses were dissatisfied with the process of utilizing SBAR. Various nurses were also inconsistent in giving the information on the plan of care, allergies, and comorbidities [27]. Alizadeh-risani et al. also faced the same scenario, not to mention that the altered model of the handover encountered the initial opposition of nurses, which could be countered by constant training and the influence of a higher degree of authority [28]. Such situations can be regarded as an illustration of the need to consider behavioral and cultural factors in the implementation strategy. Changing management strategies such as capacity building and involvement in design participation to enhance the involvement of the front line, practical support, and a feedback loop is central to the development of acceptance and, eventually, long-term use of systematic documentation practice.

Outcome Measures

Evaluation of effectiveness: The effectiveness of the modular communication models SBAR, SOAP, and PIE is greatly investigated and tested with reference to quantitative and qualitative criteria. This type of evaluation is usually used to measure the enhancement of the accuracy of other documentation, compliance with clinical protocols, and interprofessional communication. As an illustration, Tompkins proved that the use of an improved version of an SBAR tool, one that was enriched through safety checklists, led to statistically significant increases in the completeness of documentation, adherence to safety-related indicators, and interdisciplinary collaboration (p < 0.001) [30]. Similarly, Mousavi et al. also recorded the effect of SOAPIE training with a structured nursing notation checklist and found the quality of documentation improved significantly, with 46.66% documentation quality rising to 91.53% (p = 0.001) [22]. These results verify that formal communication systems not only are involved in providing consistency in documentation but also improve the overall quality and proper coordination of delivery.

Reduction in errors: The effectiveness of using structured documentation frameworks has been directly linked to the reduction of clinical errors, especially those errors that arise because of miscommunication during handoffs in various studies. Improvement of safety based on the correct exchange of information data after the introduction of SBAR excelled in an anesthetic clinic. Randmaa et al. reported that the incidence of incident reports on the ability to communicate error cases fell significantly after the introduction of SBAR into practice (p = 0.001) [24]. Abbaszade et al. found similar rises in the quality of patient care on both psychosocial and physical levels and communication when the SBAR-based handoffs were implemented [31]. This translated to higher QUALPACS scores and better rapport between the nurses and patients, implying that fewer errors and omissions were made, as far as managing patients is concerned. All these findings support the idea that structured communication models are proven to be effective safeguards against preventable adverse events through the guarantee of completeness, clarity, and consistency in transferring clinical information.

Framework Comparison

Strengths and weaknesses:** **The SBAR, SOAP, and PIE communication structures all carry distinct benefits and drawbacks based on their application in a clinical context as well as the application that any user or practitioner uses. The SBAR is especially applicable in written and verbal patient handoffs and in the event of emergency encounters. The studies by Randmaa et al. and Tompkins claimed that the accuracy of communication, the safety climate, and the completeness of documentation were substantially increased after the introduction of SBAR [24,30]. But, because of a lack of proper training, SBAR also may be deemed as a time-consuming process or lacking in more complex cases [27]. In contrast to that, SOAP fits better with detailed clinical documentation and helps diagnostic reasoning by organizing information about a patient in a logical fashion. There is a need to explore the evidence, but the most effective is to use a hybrid SBAR-SOAP format for the medically complex patients, particularly in critical care areas (Anesthesia, ER, ICU). Mousavi et al. and Sudarsan et al. discovered the benefit that SOAP enhanced the quality of nursing notes and identification of drug-related problems but associated it with limitations of linearity and time requirements. Furthermore, SOAP is more associated with physician/advanced practice provider (APP) usage in the past, which may be some of the resistance to using it. However, when SOAP is used properly, it provides a substantially better means of monitoring multiple problems and their intervention/management [22,23]. Alternatively, PIE is more concentrated on the nursing care practice and has received credit regarding the improved documentation structure and completeness. Almasi et al. proved that PIE greatly enhanced the documentation rate of nursing students, and Skingley et al. explained that barriers to the sustained use (such as staff shortage and leadership gaps) may complicate the implementation [26,35].

Best practices: These frameworks have a number of best practices found in the literature across them. First, personalized training, such as bedside coaching, simulations, and SIM, was always associated with better adoption and documentation results; refer to the works by Mousavi et al. and Almasi et al. Second, contextual adaptation, including staff, patients, and families in the development of these tools, led to increased buy-in and usability [22,26]. This could be witnessed in Velji et al. when an adapted SBAR tool had better safety culture and communication [25]. Third, compliance and efficiency were supported by integrating with electronic systems and digital tools, and the invention of workflow in tools was reported to improve documentation accuracy [27,30]. Fourth, resistance and resource limits were necessitated by powerful leadership and institutional support to withstand the obstacles. Research related to the works by Skingley et al. and Alizadeh-risani et al. highlighted the significance of a managerial engagement and organizational sustainability [28,35]. Finally, constant assessment and feedback systems, including frequent safety checks, employee surveys, and audits, ensured the continued improvements and informed any necessary changes. Collectively, the practices serve to illustrate that the effectiveness of any documentation system is not down to the design of the system itself but strategic implementation according to clinical realities.

Clinical Implications and Clinical Efficacy

The implementation of the structured communication structure (SBAR, SOAP, and PIE) has significant clinical implications to enhance patient safety, quality care, and effective communication. The tools make handoff and documentation of patient information accurate and consistent, decreasing handoff and documentation errors associated with communication. As an example, the SBAR application in the clinical setting resulted in increased accuracy of communication and fewer incident reports associated with miscommunication [24]. On a similar note, Tompkins witnessed a noteworthy improvement in documentation completeness, interdisciplinary cooperation, and safety metrics compliance after implementing a new iteration of an SBAR tool [30]. The clinical utility of SOAP and its variations was also reported; according to Mousavi et al., SOAPIE training positively changed the quality of nursing documentation (from 46.66% to 91.53%; p = 0.001), whereas A-SOAP improved drug-related problem identification rates among pharmacy students [22,23].

PIE, which is mainly applicable in nursing care settings, was reported to improve the quality, organization, and content of the nursing records. Both Almasi et al. and Rasoulzadeh et al. noted very significant differences in documentation quality between students with PIE training and control. The outcome of these improvements, clinically, is an improved continuity of care, earlier identification of patient needs, and improved multidisciplinary communication [26,29]. In addition, works, such as Abbaszade et al., showed a rise in the overall care quality, including psychosocial and physical care dimensions, that occurred as a result of taking the structured use of SBAR during handoffs [31]. Nonetheless, the level of clinical effectiveness differed with the fidelity of implementation, staff training, and organizational support. In the practices that used structured instruments that adjust to the local requirements, leadership support, and integration into the daily practice, the results were quite more effective [25,28]. Evidence gained as a combination of these results points to the clinical efficacy of the structured documentation models to optimize communication and minimize errors and patient care outcomes across the different healthcare environments.

Discussion

Randmaa et al. and Tompkins demonstrated meaningful reductions in communication-related incidents and improved safety climate after SBAR adoption [24,30]. These results align closely with a robust PubMed-indexed pre-post study across 16 hospital wards, which showed an increase in complete SBAR usage from 4% to 35% (p < 0.001), improved nurse-physician collaboration scores, a drop in unexpected deaths from 0.99 to 0.34 per 1,000 admissions, and increased detection of deterioration via unplanned ICU admissions. Supporting this, Müller et al., in their systematic review, found moderate evidence that SBAR reduces adverse events during team communications [37]. Together, these findings underscore SBAR's efficacy in enhancing clinical vigilance and reducing preventable harm. However, SBAR is presented as highly effective in improving safety and reducing errors; the majority of included studies were small, single-center, and non-randomized. Therefore, further randomized studies are required to further validate these findings. 

Current research shows that hands-on, structured training helps teams communicate better and use tools like SBAR more effectively. These matches with findings of Raurell-Torredà et al. have found that when healthcare workers practice SBAR in role-play with people from different professions (like nurses, doctors, etc.), it helps them speak more clearly, understand their own roles better, and feel more confident [38]. Beckett and Kipnis also found that SBAR training improves teamwork, makes staff more satisfied, and helps them feel that the care is safer [39]. Overall, these results show that active learning methods like practicing with simulations or role-plays are very helpful for making SBAR a normal part of teamwork in real healthcare settings.

Mousavi et al. show that using structured communication tools like SBAR makes medical records more complete and helps hospital staff work more efficiently [22]. Supporting this, studies by Panesar et al. found that in emergency and hospital care, SBAR made staff handovers faster, going from about 53 minutes to 41 minutes on paper and even faster (38 minutes) when done electronically. It also helped doctors review patients more quickly, from around two minutes to just under one minute, without lowering care quality [40]. This proves that tools like SBAR not only make communication safer but also help save time, which is very important in busy hospital settings.

Malekpour et al. in their study found that a three-week SOAPIE training significantly improved nursing documentation scores. Gianino et al. reported that SOAP fosters critical thinking by helping healthcare providers organize and differentiate clinical observations logically [33,41]. Although effective, SOAP can be time-consuming and rigid, particularly for new or overwhelmed staff. The A-SOAP format, designed for clinical pharmacy use, was shown by Sudarsan et al. to significantly enhance the identification of drug-related problems. Similar findings are supported by studies such as those by Tolley et al., who found that structured documentation tools reduce medication errors and improve therapeutic decision-making [23,42].

The PIE format focused on nursing documentation [26,29]. These studies showed that PIE improved documentation structure and clarity among nursing students. Supporting evidence from Kovach et al. found that PIE facilitates continuity of care, reduces duplication, and improves accountability in nursing practice [43]. Another study, Papathanasiou et al., confirmed that PIE increases documentation quality while decreasing time spent on charting [44]. However, our findings and prior studies alike noted that PIE implementation can be limited by organizational readiness, lack of training, and leadership engagement, as seen in Skingley et al. [35].

Limitations and Recommendations

All the studies conducted and included shared some major limitations, which impair the generalizability and usefulness of the investigated evidence in the long term. Many of them used quasi-experimental designs that lack any restrictions that assure validity, exposing them to a higher likelihood of a confounding factor accompanied by selection. The sample sizes of many studies were small, and often, it was problematic to make a definite conclusion on a larger population. Follow-up intervals were short (usually less than one month), so it was not possible to evaluate the long-lasting effect on the quality of documentation and outcomes of patients. In most instances, data that has been reported by self or audits without blind assessment presented the risk of reporting bias. Moreover, it could not be fully implemented and assimilated in places and organizations due to the development of obstacles and weaknesses at both organizational and contextual levels. The future studies of these problems should focus on the randomized controlled trials that are conducted with large and multi-centered samples, and they should have long follow-up times to assess the enduring changes within practice and patient safety. To this end, resistance and subsequent compliance could be addressed through the development and testing of hybrid models (i.e., a combination of SBAR with digital checklists or bedside documentation). Moreover, investigators ought to integrate a mixed-method strategy where quantitative results should be used along with qualitative feedback from both the staff and patients as far as usability, feasibility, and obstructions are concerned. These tools also need effective mechanisms to scale them across different healthcare facilities, and this requires strong institutional support, effective leadership involvement, and training infrastructure.

## Conclusions

The review concluded that SBAR, SOAP, and PIE have the potential to foster documentation, minimize communication mistakes, and focus on care quality, especially when entwined with teaching, motive, and online synchronization. However, the evidence was synthesized from the moderate-quality studies (due to high heterogeneity) with small sample sizes and based on single centers, which warrants further validation through high-quality studies (e.g., randomized studies). Nonetheless, the various gaps in the area of long-term effects, scalability, and implementation fidelity identify the necessity of high-quality, multi-center, randomized, and controlled trials lasting longer periods with the outcomes of real-life condition monitoring.
